# Epidemiological Investigation on Pathogenic Bacteria of Buffalo Subclinical Mastitis and Their Antibiotic Resistance and Virulence Characteristics in Guangxi, China

**DOI:** 10.3390/ani15223321

**Published:** 2025-11-18

**Authors:** Ling Li, Jiaping Zhang, Xingqi Wei, Ruimin Wang, Xia Dan, Jianfeng Li, Enghuan Hau, Qingkun Zeng, Qingyou Liu, Jiafeng Ding, Kuiqing Cui

**Affiliations:** 1 College of Animal Science and Technology, Guangxi University, Nanning 530004, China; lling2010@163.com (L.L.); weixingqi24@163.com (X.W.); l2240990512@163.com (J.L.); qyliu-gene@fosu.edu.cn (Q.L.); 2 Guangxi Zhuang Autonomous Region Buffalo Milk Quality and Safety Control Technology Engineering Research Center, Guangxi Buffalo Research Institute, Chinese Academy of Agricultural Sciences, Nanning 530001, China; zhangjiaping1997@163.com (J.Z.); ruimin0708@126.com (R.W.); 17807889919@163.com (X.D.); enghuan_90@yahoo.com (E.H.); zqk456@163.com (Q.Z.); 3 Guangdong Provincial Key Laboratory of Animal Molecular Design and Precise Breeding, School of Life Science and Engineering, Foshan University, Foshan 528225, China

**Keywords:** subclinical mastitis, pathogenic bacteria, antibiotic resistance, virulence, buffalo

## Abstract

Subclinical mastitis (SCM) is one of the most common and detrimental diseases affecting dairy cows, causing lower milk yield and quality. Identification of pathogenic bacteria of subclinical mastitis (PSM) in dairy cows is crucial for selecting suitable antibiotic treatments and implementing effective prophylactic measures. This paper highlights the first systematic study to investigate the PSM in buffalo farms in Guangxi, China. It also analyzes the antibiotic resistance and virulence characteristics of typical PSM. The bacteriological characteristics of 132 milk samples collected from buffaloes with SCM across 3 representative buffalo farms in Guangxi, China were investigated. A total of 1659 bacterial strains were isolated and classified into 46 genera and 183 species, where 1058 bacterial strains were identified as PSM, representing 64% of the total isolates. The frequently isolated PSM in total samples were coagulase-negative *Staphylococci* (55.30%), *Enterococcus faecalis* (51.52%), *Escherichia coli* (31.82%), and *Klebsiella pneumoniae* (28.03%). All PSM strains showed multiple antibiotic resistance. Notably, *E. faecalis* and *Lactococcus garvieae* were resistant to all 12 antibiotics, whereas *Staphylococcus chromogenes* (95.24%), *E. coli* (89.19%), *K. pneumoniae* (83.87%), and *Staphylococcus epidermidis* (83.33%) were sensitive to levofloxacin (LEV). Additionally, *E. coli* exhibited the strongest mortality of *Galleria mellonella*. This study concluded that multiple PSM are present in the milk of buffaloes suffering from SCM in Guangxi, China. LEV may be a suitable antibiotic for the treatment of PSM. In the future, it is necessary to monitor the prevalence of PSM in buffalo farms and develop control strategies to prevent their spread.

## Simple Summary

Subclinical mastitis (SCM) is a major but often neglected issue in dairy farming, affecting milk yield and quality. Identification of pathogenic bacteria of subclinical mastitis (PSM) in dairy cows is crucial for implementing effective prophylactic and control measures. This paper highlights the first systematic study to investigate the PSM in buffalo farms in Guangxi, China. It also analyzes the antibiotic resistance and virulence characteristics of typical PSM. A total of 1659 bacterial strains were isolated from 132 milk samples with SCM, among which 1058 were identified as PSM. Coagulase-negative *Staphylococci* (55.30%), *Enterococcus faecalis* (51.52%), *Escherichia coli* (31.82%), and *Klebsiella pneumoniae* (28.03%) were frequently isolated PSM in total samples. All PSM strains showed multiple-antibiotic resistance. *E. faecalis* and *Lactococcus garvieae* were resistant to all 12 antibiotics. *E. coli* exhibited the strongest mortality of *Galleria mellonella*. These results highlight that the prevention and control of PSM in buffalo farms should be strengthened.

## 1. Introduction

Buffalo milk is the second most consumed milk globally, second only to dairy cow milk [1]. It contains higher levels of protein, total solids, and vitamins compared to dairy cow milk [2]. Subclinical mastitis (SCM) is one of the most prevalent and damaging diseases affecting dairy animals. Apart from reducing milk yield and quality, SCM also increases the cost of breeding and processing, impedes the development of the dairy sector and poses potential risks to public health [3,4,5]. SCM is defined as an elevated level of milk somatic cell count (SCC), at which raw milk from healthy dairy cows has less than 1.5 × 10^5^/mL SCC [6,7].

The pathogenesis of SCM in dairy cows is complex, with pathogenic bacterial infections serving as its main contributing factors [8]. Many bacterial species have been linked to bovine mastitis (Supplementary Table S1), including coagulase-negative *Staphylococci* (CoNS) [9], *Staphylococcus aureus* [10], *Klebsiella pneumoniae* [11], *Escherichia coli*, *Macrococcus caseolyticus* [12], *Lactococcus garvieae* [13] and others. CoNS is the primary pathogen causing SCM in dairy cows, including *Staphylococcus chromogenes*, *Staphylococcus haemolyticus*, *Staphylococcus epidermidis*, *Staphylococcus simulans*, and *Staphylococcus xylosus* [9,14,15]. It is worth noting that not all CoNS are pathogenic bacteria of subclinical mastitis (PSM), and no studies have shown that *Staphylococcus cohnii*, *Staphylococcus capitis*, and *Staphylococcus edaphicus* belong to PSM.

There are obvious regional differences in the distribution of buffalo PSM. *Staphylococcus* spp. and *E. coli* are the most commonly isolated buffalo PSM, followed by *Streptococcus* spp. and *Klebsiella* spp. in northwestern Pakistan [4]. On the other hand, *Staphylococcus* spp. are the most prevalent and commonly isolated PSM strains, followed by *E. coli*, *Pseudomonas* spp., and *Bacillus* spp. in the Punjab province of Pakistan [16]. A survey in 2007 showed that the detection ratio of *E. coli* and *S. aureus* in raw buffalo milk samples from Guangxi and Yunnan provinces of China was high, but PSM was not isolated for targeted research [17]. China is one of the world’s leading producers of buffalo milk, with the primary production area in Guangxi Province [17]. Nevertheless, there is limited data on the specific pathogens affecting buffaloes in this region.

Antibiotic resistance in bacteria becomes more and more challenging [4,18,19]. Most buffalo PSM were resistant to sulfamethoxazole (99.3%), lincomycin (98%), oxytetracycline (89.3%), and ampicillin (AMP, 86.1%) in Northwest Pakistan [4]. An antibiotic resistance test of *E. coli* and *K. pneumoniae* from buffalo in Bangladesh showed that these strains revealed significant resistance to AMP, amoxicillin-clavulanic acid, and aminoglycosides, with 31.5% of *E. coli* and 39.3% of *K. pneumoniae* isolates exhibiting multiple antibiotic resistance [18]. It is worth noting that the virulence of PSM is also severe. The mortality of *Galleria mellonella* larvae was 60% after 48 h of infection with *E. coli* [20]. On the other hand, 75% of *G. mellonella* larvae died after 24 h of infection with *K. pneumoniae*, while the remaining survivors died within 72 h [21]. Hence, it is essential to develop effective strategies to reduce the spread of pathogens in cattle herds within particular geographic areas by understanding the population structure, spread, virulence traits, and antibiotic resistance of PSM. It is worth noting that there are few systematic studies on the antibiotic resistance and virulence of Chinese buffalo PSM.

This study aimed to investigate the prevalence of PSM in buffalo farms in Guangxi, China. The antibiotic resistance and virulence of typical PSM were further analyzed by the disk diffusion method and *G. mellonella* larvae infection test. This study will provide an important basis for the prevention and treatment of buffalo SCM in Guangxi.

## 2. Materials and Methods

### 2.1. Collection of Samples

Three representative buffalo farms were investigated in Guangxi, China. The farms were labeled as Herd A, Herd B, and Herd C. Herd A is the major buffalo breeding farm in China, comprising purebred Murrah buffalo, purebred Nile/Rafi buffalo, and a variety of hybrid dairy buffalo. Herds B and C were large commercial buffalo farms that primarily raised hybrid dairy buffaloes.

Previous studies have shown that SCM increases the SCC in milk [6,7]. In healthy cows, milk SCC is typically below 1.5 × 10^5^/mL, and some even below 1 × 10^5^/mL [6,22]. Based on this standard, milk samples with SCC greater than 1.5 × 10^5^/mL are classified as coming from SCM cows.

Raw buffalo milk samples were collected randomly from three representative buffalo farms in the morning and afternoon from March 2023 to January 2024. Sample collection and procedures were performed according to the steps of Ranasinghe et al. [23]. The udder was disinfected with 1.0% (*m*/*v*) povidone iodine before sampling. Then the first three streams of milk were discarded, followed by buffalo milk samples collected in sterile tubes and stored at 4 °C. The SCC analysis was conducted within 2 h using Bacsomatic^TM^ (FOSS, Hillerød, Denmark), strain isolation was performed on SCM milk samples within 24 h of sampling.

### 2.2. Isolation of Bacteria

**Figure 1:**
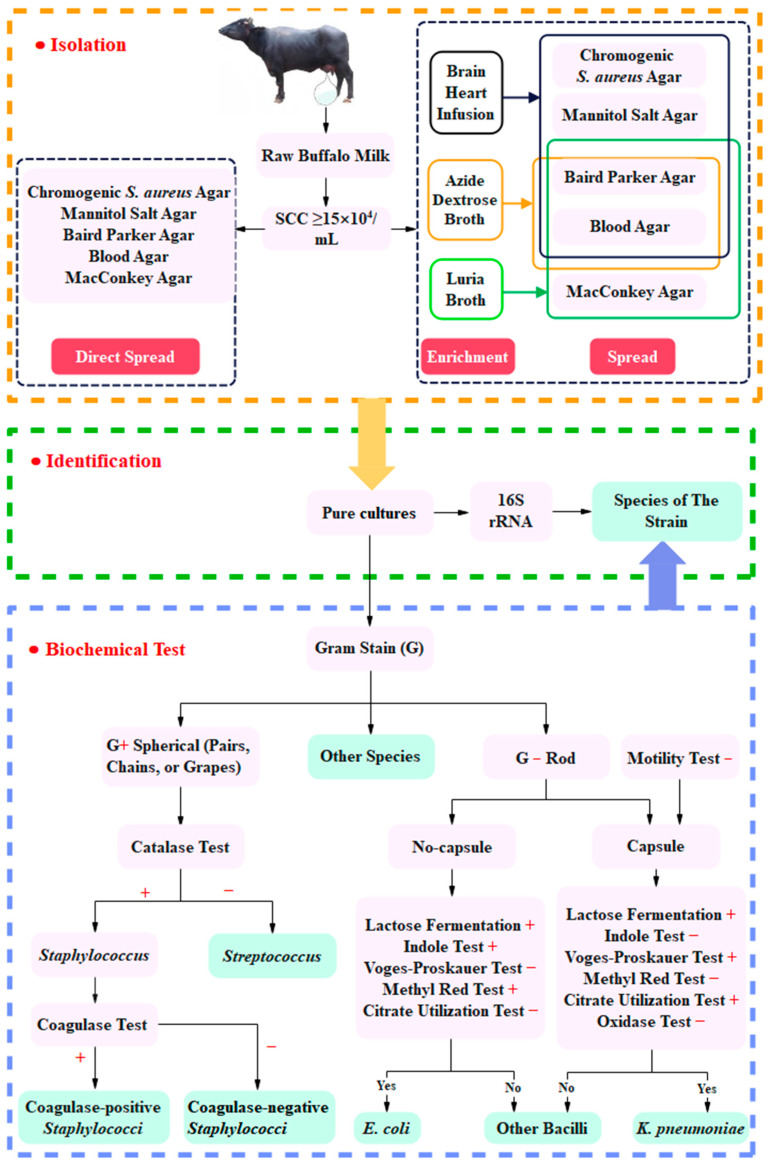
Isolation and identification process of PSM from buffalo milk. + and − indicate positive and negative results for the indicators, respectively.

There were two methods applied to separate PSM: (a) direct spread [11,24] and (b) enrichment before spread [25,26,27]. The separation process is shown in Figure 1.

(a) Direct spread: 0.1 mL of milk samples was directly spread on chromogenic *S. aureus* agar, mannitol salt agar, Baird Parker agar, blood agar, and MacConkey agar. Then, plates were incubated at 37 °C for 24 h to 48 h.
(b) Enrichment before spread: 5 mL of milk samples was added to 45 mL of brain heart infusion, azide dextrose broth, and Luria broth, respectively, and cultured at 37 °C for 18 h to 24 h. One milliliter of cultures was added into a sterile test tube having 9 mL of sterile water. After mixing, the culture was serially diluted up to 1: 10^5^. Then, 0.1 mL of each dilution was spread to corresponding agar plates (Figure 1) and incubated at 37 °C for 24 h to 48 h. The culture enriched by brain heart infusion was spread on chromogenic *S. aureus* agar, mannitol salt agar, Baird Parker agar, and blood agar. The culture enriched by azide dextrose broth was spread on Baird Parker agar and blood agar. The culture enriched by Luria broth was spread on Baird Parker agar, blood agar, and MacConkey agar.

Single colonies picked from the above plates were streaked on tryptic soy agar to prepare pure cultures. All culture media were purchased from Qingdao Haibo Biology Company (Qingdao, China).

### 2.3. Identification of Pathogenic Bacteria of Subclinical Mastitis

The biochemical and 16S ribosomal ribonucleic acid (16S rRNA) of the strain were determined based on the microbial identification procedure described in Figure 1 [11], in order to determine the PSM species. The biochemical tests flowchart in Figure 1 illustrates the identification process for coagulase-positive *Staphylococci*, CoNS, *E. coli*, and *K. pneumoniae*. In detail, coagulase-positive *Staphylococci*, CoNS were confirmed by gram stain, catalase test, and coagulase test [28]. *E. coli* and *K. pneumoniae* were confirmed by Gram-stain, capsular staining, lactose fermentation, indole, Voges-Proskauer, methyl red, and citrate utilization tests [24,29].

The well-established method was utilized to extract deoxyribonucleic acid (DNA) from PSM as previously described by de Boer et al. [30]. Further, the extracted DNA was used for polymerase chain reaction (PCR) amplification by the ProFlex PCR System (Life Technologies, Foster City, CA, USA). The reaction was prepared as follow; 12.5 µL of 2× Super Flash Master Mix (Kangwei Century, Taizhou, China), 0.5 µL of 27F (AGAGTTTGATCMTGGCTCAG), 0.5 µL of 1492R (GGTTACCTTGTTACGACTT), 0.5 µL of extracted DNA, and 11 µL of double distilled water. Primers 27F and 1492R were biosynthesized by Beijing Qingke Biotechnology Co., Ltd. (Beijing, China). Amplification consisted of one cycle of initial denaturation at 95 °C for five minutes, followed by 34 cycles of denaturation at 94 °C for 60 s, annealing at 55 °C for 45 s, and extension at 72 °C for 90 s; and a final extension cycle at 72 °C for ten minutes. The 16S rRNA was determined by Beijing Qingke Biotechnology Co., Ltd. (Beijing, China) and compared by BLAST in NCBI website (https://blast.ncbi.nlm.nih.gov/Blast.cgi (accessed on 30 January 2024)).

A list of reported PSM isolates has been collected (Supplementary Table S1). The strains isolated in this study were compared with the Supplementary Table S1 to determine whether our isolates were PSM.

### 2.4. Screening Principles for Strains Used in Antibiotic Resistance and Galleria mellonella Larvae Infection Tests

The typical PSM were selected as the research subjects to investigate the antibiotic resistance and virulence characteristics of buffalo PSM in Guangxi, China. Total sample isolation ratios of typical PSM were more than 20% (Table 1), including *Enterococcus faecalis*, *K. pneumoniae*, *E. coli*, *L. garvieae*, and CoNS (*Staphylococcus chromogenes* and *Staphylococcus epidermidis*). The 16S rRNA phylogenetic tree of 6 typical PSM was constructed by MEGA 11, and the clustering was carried out according to the standard of 16S rRNA gene sequence similarity ≥ 99%. In each cluster, only individual strains isolated from the milk samples of the same buffalo at the same time were retained. After the above screening, 147 representative strains were finally screened from 6 typical PSM for subsequent antibiotic *resistance* and *G. mellonella* larvae infection tests. The numbers of strains tested were as follows: 29 *E. faecalis*, 31 *K. pneumoniae*, 37 *E. coli*, 21 *S. chromogenes*, 11 *L. garvieae*, and 18 *S. epidermidis* (Table 1).

### 2.5. Analysis on Antibiotic Resistance of Typical PSM

The disk diffusion method was used to perform antibiotic resistance testing on Mueller-Hinton agar plates in accordance with the guidelines established by the Clinical and Laboratory Standards Institute [31].

Based on the veterinary antibiotic list of the World organization for Animal Health (https://www.woah.org/en/document/list-of-antimicrobial-agents-of-veterinary-importance/ (accessed on 29 July 2024)), twelve veterinary antibiotics spanning nine different antibiotic classes were selected for antibiotic resistance test. The following antibiotics purchased from Macklin (Shanghai, China) were used: penicillin (ampicillin 10 µg, penicillin 30 IU), aminoglycosides (amikacin 30 µg, gentamicin 30 µg), quinolones (levofloxacin 30 µg, ciprofloxacin 5 µg), cephalosporins (ceftriaxone 30 µg), tetracycline (tetracycline 30 µg), β-lactam (amoxicillin 20 µg), macrolides (azithromycin 15 µg), chloramphenicol (chloramphenicol 30 µg), and folate pathway antagonists (trimethoprim 10 µg). The antibiotic resistance of the strain was categorized as sensitive (S), intermediate (I), or resistant (R), based on the inhibition zone diameters on the plate.

### 2.6. Analysis on Virulence of Typical PSM

*G. mellonella* larvae have emerged as a useful insect model in research on host–pathogen interactions [32], and the reliability of the larvae infection tests on PSM (*E. faecalis*, *K. pneumoniae*, *E. coli*) virulence have been validated by several investigations [33,34,35]. Hence, the current study also applied the similar method to test on the virulence of typical PSM (Laughing Monkey Information Technology Company, Chongqing, China).

The appropriate colony concentration was selected according to the previous research and pre-experimental results [21,36,37,38], injected into the larvae of *G. mellonella*. The experimental concentrations of *E. coli* [36] and *K. pneumoniae* [21] were 10^3^ CFU/larvae, those of *S. chromogenes* [37], *L. garvieae* [37], and *S. epidermidis* [37] were 10^4^ CFU/larvae, and that of *E. faecalis* [38] was 10^5^ CFU/larvae. Bacterial suspensions (10 µL) were inoculated on the last left worm proleg, and the *G. mellonella* larvae were incubated at 37 °C (negative controls were only inoculated with 10 µL saline). Each strain was inoculated into 10 *G. mellonella* larvae, the mortality of *G. mellonella* larvae were observed and recorded every 12 h.

### 2.7. Data Processing

Excel spreadsheets were used for meticulous compilation and classifying the data. SPSS version 26 was used to perform all the statistical analyses. Origin 2025 was used to draw all figures. Adobe Illustrator 2024 was used for figures retouching.

## 3. Results

### 3.1. Prevalence and Isolation of Buffalo PSM in Different Farms

**Figure 2:**
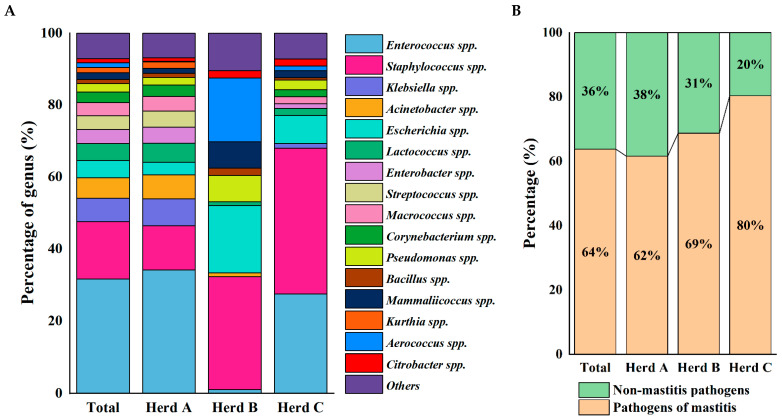
(**A**) Bacterial isolation of milk samples from buffaloes with subclinical mastitis across different farms at genus level. (**B**) The separation ratio of pathogenic bacteria of subclinical mastitis in each farm. The numbers of strains isolated from total, Herd A, Herd B, and Herd C were 1659, 1410, 96 and 153, respectively.

Bacteriological examination was performed on 132 milk samples collected from buffaloes with SCM across 3 representative buffalo farms in Guangxi, China. A total of 1659 bacterial strains encompassing 183 species across 46 genera were identified based on biochemical and 16S rRNA analysis (Supplementary Table S2). As shown in Figure 2A, *Enterococcus* spp. (31.65%) was the most isolated genera in total samples, followed by *Staphylococcus* spp. (15.97%). The dominant genera were *Enterococcus* spp. (34.18%) and *Staphylococcus* spp. (12.27%) in Herd A, while *Staphylococcus* spp. (31.25%), *Escherichia* spp. (18.75%), and *Aerococcus* spp. (17.71%) in Herd B. In contrast to Herd A, *Staphylococcus* spp. (40.52%) was more dominant, followed by *Enterococcus* spp. (27.45%) in Herd C.

A total of 1058 pathogenic bacteria of subclinical mastitis (PSM) were identified (Supplementary Tables S1 and S2). PSM accounted for over 62% of all the isolates across the three herds, with Herd C having the highest PSM isolation ratio, representing 80% of all the isolates (Figure 2B).

**Figure 3:**
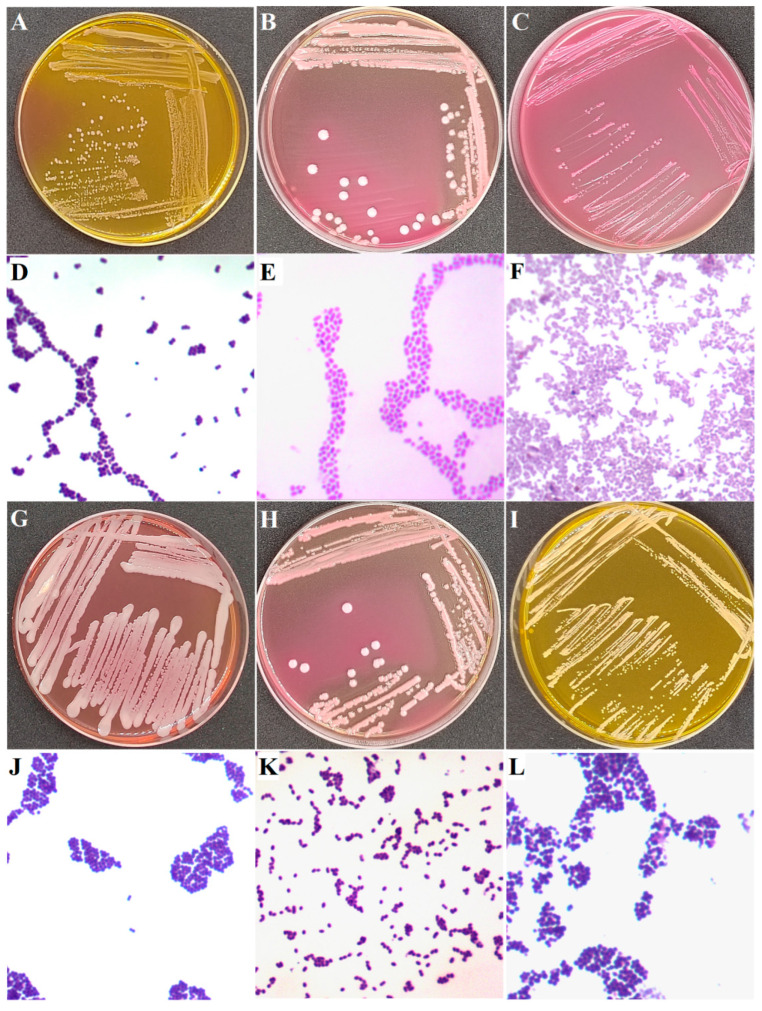
Colony morphology and Gram-staining (100×) of typical PSM. (**A**) Colony morphology of *E. faecalis*, (**B**) Colony morphology of *K. pneumoniae*, (**C**) Colony morphology of *E. coli*, (**D**) Gram staining of *E. faecalis*, (**E**) Gram staining of *K. pneumoniae*, (**F**) Gram staining of *E. coli*, (**G**) Colony morphology of *S. chromogenes*, (**H**) Colony morphology of *L. garvieae*, (**I**) Colony morphology of *S. epidermidis*, (**J**) Gram staining of *S. chromogenes*, (**K**) Gram staining of *L. garvieae*, (**L**) Gram staining of *S. epidermidis*.

Strains with an isolation ratio of more than 20% in total samples were regarded as typical PSM (Table 1), including *Enterococcus faecalis*, *Klebsiella pneumoniae*, *Escherichia coli*, *Staphylococcus chromogenes*, *Staphylococcus epidermidis*, and *Lactococcus garvieae*. Figure 3 shows the colony morphology and Gram-staining results of typical PSM. As shown in Table 1, *E. faecalis* was the most frequently isolated strains. A total of 386 *E. faecalis* strains were isolated from 68 samples, which were mainly distributed in the samples of Herd A (52.17%) and Herd C (88.89%). In addition, 85 strains of *K. pneumoniae* were isolated from 37 samples, which were isolated solely from samples of Herd A (32.17%). A total of 75 strains of *E. coli* were isolated from 42 samples, which were distributed in the samples of Herd A (24.35%), Herd B (75.00%), and Herd C (88.89%). Next, 58 strains of *L. garvieae* were isolated from 31 samples, which were isolated from samples of Herd A (26.09%) and Herd C (11.11%). Notably, 257 strains of CoNS were isolated from 73 samples, and 197 CoNS strains were PSM. No studies have shown that another 60 CoNS strains are associated with subclinical mastitis in dairy cows. The main CoNS were *S. chromogenes*, and a total of 59 *S. chromogenes* strains were isolated from 25 samples from Herd A (13.04%), Herd B (25.00%), and Herd C (88.89%).

*Macrococcus caseolyticus* (*n* = 59), *Enterobacter cloacae* (*n* = 33), *Mammaliicoccus sciuri* (*n* = 29), *Staphylococcus haemolyticus* (*n* = 28), *Staphylococcus borealis* (*n* = 24), and *Aerococcus viridans* (*n* = 20) were also frequently isolated PSM. In addition, *Acinetobacter baumannii* (*n* = 42), *Enterococcus gallinarum* (*n* = 40), *Streptococcus macedonicus* (*n* = 25), *Kurthia gibsonii* (*n* = 25), and *Staphylococcus cohnii* (*n* = 20) were not PSM, but their isolated strains were also greater than 20.

### 3.2. Antibiotic Resistance of Typical PSM

**Figure 4:**
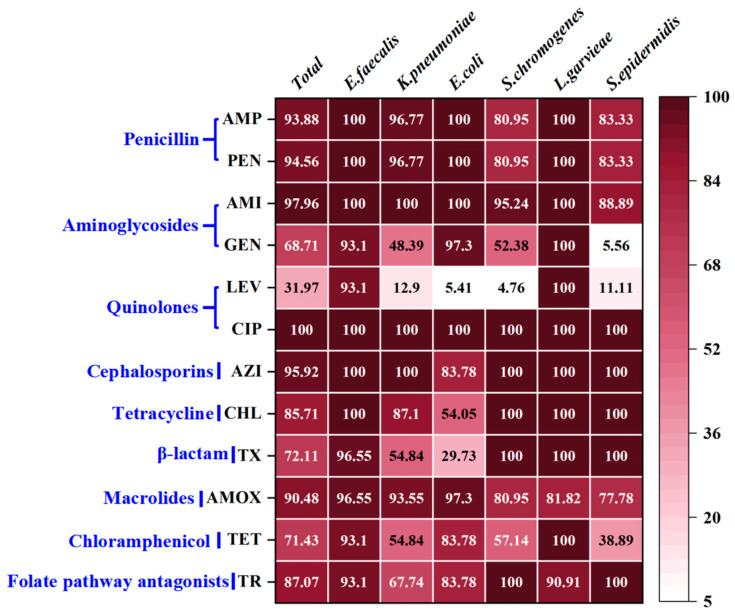
The antibiotic resistance of typical PSM. The deeper the red, the stronger the antibiotic resistance. Numbers indicate the percentage (%) of antibiotic-resistant strains in the strain. Blue fonts represent antibiotic classes. AMP, ampicillin; PEN, penicillin; AMI, amikacin; GEN, gentamicin; LEV, levofloxacin; CIP, ciprofloxacin; AZI, azithromycin; CHL, chloramphenicol; TX, ceftriaxone; AMOX, amoxicillin; TET, tetracycline; TR, trimethoprim.

**Figure 5:**
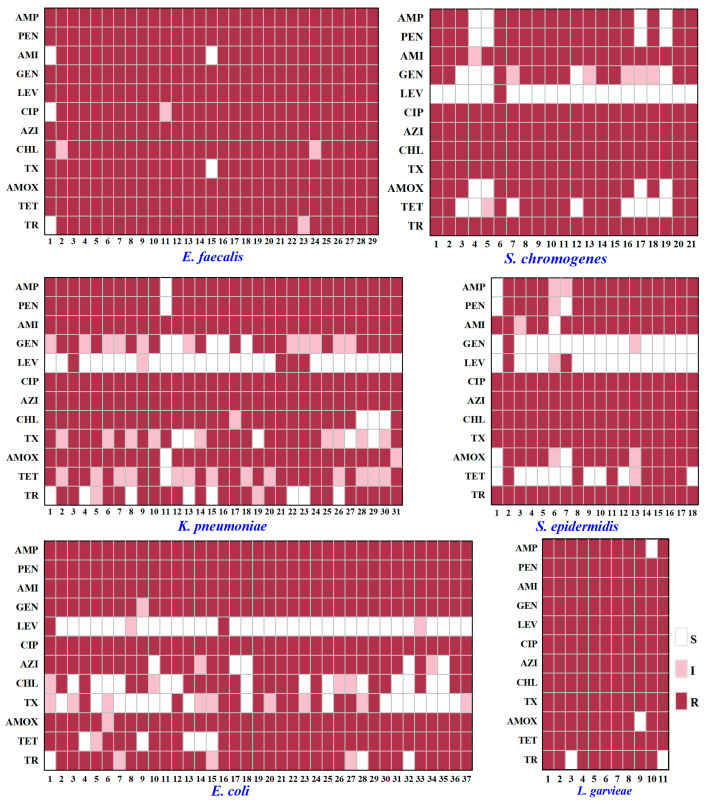
Characteristics of antibiotic resistance of 147 representative PSM strains. The abscissa shows the strain number. S, sensitive; I, intermediate; R, resistant; AMP, ampicillin; PEN, penicillin; AMI, amikacin; GEN, gentamicin; LEV, levofloxacin; CIP, ciprofloxacin; AZI, azithromycin; CHL, chloramphenicol; TX, ceftriaxone; AMOX, amoxicillin; TET, tetracycline; TR, trimethoprim.

**Figure 6:**
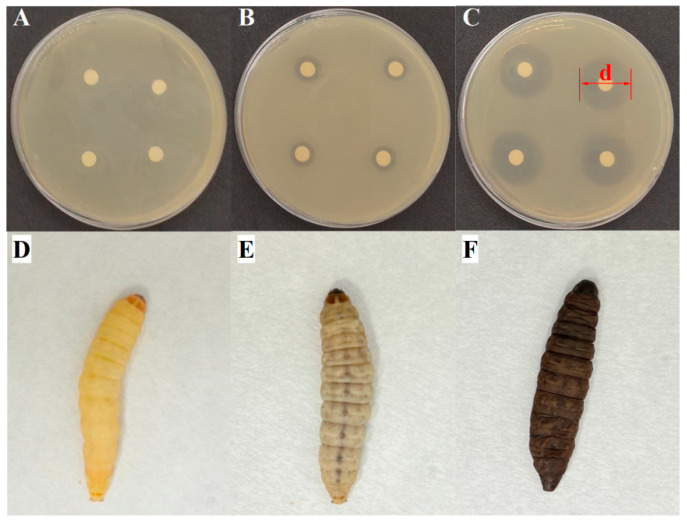
Phenomenon of antibiotic susceptibility and *Galleria mellonella* larvae infection tests. (**A**) resistant strain, (**B**) intermediate strain, (**C**) sensitive strain, (**D**) Normal *G. mellonella* larvae. (**E**) Infected *G. mellonella* larvae. (**F**) Dead *G. mellonella* larvae. d indicates the inhibition zone.

As shown in Figure 4, significant resistance was observed in six typical PSM against ciprofloxacin (CIP, 100%), amikacin (AMI, 97.96%), and azithromycin (AZI, 95.92%). In contrast (Figure 4), *K. pneumoniae* (12.90%), *E. coli* (5.41%), *S. chromogenes* (4.76%), and *S. epidermidis* (11.11%) showed low resistance to levofloxacin (LEV). Among 147 representative PSM strains (Figure 5), all of them showed multiple antibiotic resistance (resistance to ≥3 antibiotic classes). As shown in Figure 5, *E. faecalis* and *L. garvieae* were resistant to all 12 antibiotics, whereas *K. pneumoniae* (26/31, 83.87%), *E. coli* (33/37, 89.19%), and *S. chromogenes* (20/21, 95.24%) were sensitive to LEV. *S. epidermidis* was sensitive to gentamicin (16/18, 88.88%) and LEV (15/18, 83.33%). Figure 6A–C shows the experimental phenomenon of antibiotic resistance in typical PMS.

### 3.3. Virulence of Typical PSM

**Figure 7:**
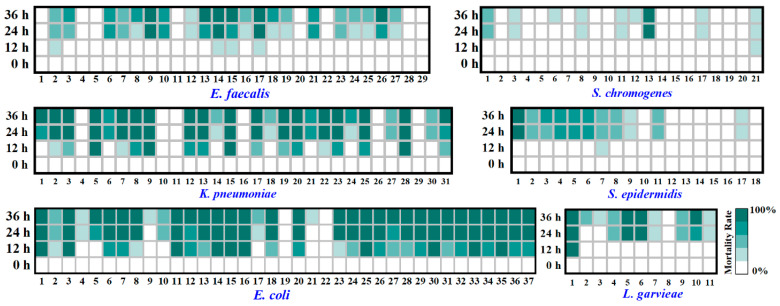
Characteristics of *G. mellonella* mortality of 147 representative PSM strains. The abscissa shows the strain number. The deeper the green color, the more mortality is caused.

*Galleria mellonella* larvae were used as an in vivo model to assess the pathogenicity of typical PSM strains. Healthy *G. mellonella* larvae was pale yellow (Figure 6D), whereas the body color gradually blackened after infection with pathogenic bacteria (Figure 6E). The body color of the dead larvae was dark black (Figure 6F). All six typical PSM showed pathogenicity against *G. mellonella* larvae (Figure 7). *E. coli* (10^3^ CFU/larvae) exhibited the strongest mortality against *G. mellonella* larvae, at which 10 out of 37 *E. coli* strains induced a mortality rate of more than 90% at 12 h post-injection, and 29 out of 37 *E. coli* strains induced a mortality rate exceeding 90% at 36 h post-injection. Moreover, 18 out of 31 *K. pneumoniae* (10^3^ CFU/larvae) strains, 4 out of 11 *L. garvieae* (10^4^ CFU/larvae) strains, and 6 out of 29 *E. faecalis* (10^5^ CFU/larvae) strains induced a mortality rate exceeding 90% in *G. mellonella* larvae at 36 h post-injection. However, only 1 out of 21 *S. chromogenes* (10^4^ CFU/larvae) strains and 1 out of 18 *S. epidermidis* (10^4^ CFU/larvae) strains induced a mortality rate exceeding 90% in *G. mellonella* larvae at 36 h post-injection.

## 4. Discussion

SCM is an important issue in the buffalo breeding industry, which will reduce milk yield and quality [4]. The occurrence of mastitis in dairy cows is complex, and pathogenic bacterial infection is one of the influencing factors [8]. In our study, *Enterococcus faecalis*, CoNS, *Klebsiella pneumoniae*, and *E. coli* were the most frequently isolated PSM, and their total sample isolation ratios were all greater than 28% (Table 1). *Staphylococcus* spp. were the most commonly isolated PSM in buffalo farms, followed by *K. pneumoniae* and *E. coli* in Pakistan [4,16], whereas *K. pneumoniae* and *E. coli* were the most commonly isolated PSM in some dairy farms in China [11,39]. Additionally, *Enterococcus* spp. showed the highest average relative abundance, followed by *Staphylococcus* spp. and *Lactococcus* spp. in a metagenomic analysis of mastitic buffalo milk samples from India [40]. Existing studies have shown that the differences in the geographical distribution of PSM are related to variety of factors, including internal factors (age, parity, lactation stage and health status) and extrinsic factors (udder hygiene, padding, milking machine, management, climate and region) [39].

*E. faecalis* is one of the most isolated PSM and is ubiquitous in the gastrointestinal tract of mammals and the environment [41,42]. In this study, 386 strains of *E. faecalis* were isolated from 132 milk samples (Table 1). Różańska et al. isolated 360 strains of *E. faecalis* from 426 milk samples suspected of mastitis [43]. However, the isolation ratios of *E. faecalis* in milk samples from the Czech Republic and northeastern Brazil were 20.9% and 26.3%, respectively [44,45]. In addition, the isolation ratio of *E. faecalis* was only 4.5% in dairy cow PSM from 62 commercial farms in 15 provinces of China (few buffalo samples) [46]. The difference in the distribution of *E. faecalis* may be associated with feces and equipment in contact with feces, the study of Lee et al. supported this speculation [47].

CoNS is the main pathogen causing SCM in dairy cows [9,14]. The mechanism of CoNS causing SCM is complex and species-specific, primarily leading to the occurrence of SCM through a multi-faceted synergistic action involving adhesion colonization, immune evasion, destruction of mammary tissue, and interference with host metabolism [48]. A total of 257 strains of CoNS were isolated in this study, of which 197 were PSM (Table 1). The CoNS species differ significantly depending on the region. *Staphylococcus chromogenes*, *S. simulans*, *S. xylosus*, and *Staphylococcus cohnii* were the major PSM associated with bovine mastitis in three dairy farms in Flanders, Belgium [49]. However, the isolation ratios of *S. chromgenes*, *S. epidermidis*, and *Staphylococcus haemolyticus* were higher in Bangladesh [50]. An extensive survey of the prevalence of CoNS in major dairy cows in China revealed that *S. chromogenes* (33%) exhibited the highest isolation ratio, followed by *S. epidermidis* (8%) [51]. Aligned to the previous literature, the current study also found that *S. chromogenes*, *S. epidermidis*, and *S. haemolyticus* were still the major CoNS in Guangxi Province, China (Table 1). Additionally, the isolation count of *Staphylococcus borealis* (*n* = 24) in this region was at an alarming level. Relevant literature shows that the use of milking machine will increase the risk of mastitis in cows [52]. This is because the milking machine will cause the nipple tube to remain open after milking, and the Staphylococcus attached to the pipe of the breast pump can easily enter the cow ‘s breast [53].

*E. coli* and *K. pneumoniae* were also typical PSM isolates in this study, with isolation ratios of 31.82% and 28.03% of total samples (Table 1), respectively. These sample isolation ratios were higher than an epidemiological survey of *E. coli* (13.38%) and *K. pneumoniae* (13.96%) in buffalo SCM samples in Bangladesh [18]. However, our results were comparable to those samples in Xinjiang, China where the isolation ratio of *K. pneumoniae* in SCM samples was 23.2%, and those samples in Romania where the isolation ratio of *E. coli* in mastitis samples was 27.51% [54]. Both *E. coli* and *K. pneumoniae* are environmental pathogens, and padding, milking machines, feces and equipment in contact with feces are their sources of pollution [55].

In the current study, *Aerococcus* spp. was the characteristic genus in Herd B (Figure 2A), where *Aerococcus viridans* was the most isolated strain of *Aerococcus* spp. (Table 1). A total of 20 *A. viridans* strains were isolated from all three herds. *A. viridans* is considered an emerging etiological agent of bovine SCM, wherein it exerts a negative effect on somatic cell count, milk yield, and composition [56]. A total of 69 strains of *A. viridans* were isolated from 1774 mastitis milk samples collected in Korea from 2016 to 2021, accounting for 3.9% of isolation counts [57]. This study is the first report on the prevalence of *Aerococcus viridans* in Chinese buffaloes.

Antibiotic resistance has emerged as an alarming concern in PSM from buffaloes [57]. In our study, 147 representative PSM strains from buffalo exhibited multiple antibiotic resistance (Figure 5), surpassing the levels of resistance in cattle reported in previous studies [4,45]. Previous literature reported that 72 strains of PSM were resistant to penicillin (PEN, 79.1%), AMP (77.7%), and tetracycline (TET, 63.8%), with 83.4% of PSM showed multidrug resistance in northeastern Brazil [46]. In contrast, most of the PSM were resistant to sulphamethoxazole (99%), lincomycin (98%), oxytetracycline (89%), AMP (86%), and doxycycline (85%) in the cattle and buffalo farms of northeast Pakistan [4].

In this study (Figure 4), *E. faecalis* and *Lactococcus garvieae* exhibit high resistance to multiple antibiotics because they are prone to transferring resistance genes, that cause widespread resistance to antibiotics [58,59]. All *E. coli* strains exhibited strong antibiotic resistance, with 100% resistance to AMP, PEN, AMI, and CIP (Figure 4), aligned to the antibiotic resistance in cow mastitis [19,60,61]. Similarly, *K. pneumoniae* also exhibited 100% resistance to AMI, CIP, and AZI, while its resistance towards AMP, PEN, and amoxicillin (AMOX) had exceeded 93% (Figure 4). Consistent with earlier findings, *K. pneumoniae* from dairy cows was typically resistant to AMP [62], chloramphenicol [63], TET [63], and AMOX [64]. What’s more, the total antibiotic resistance of *S. chromogenes* and *S. epidermidis* was similar, although some differences between individual strains were observed (Figure 4 and Figure 5). Previous studies also reported the resistance of CoNS (including *S. chromogenes* and *S. epidermidis*) to antibiotics such as PEN [65], TET [66] and AMP [67].

It is worth noting that LEV showed the highest antibiotic sensitivity to 147 representative strains (Figure 5). LEV is the third generation of quinolones, exhibiting greater activity against pathogens compared to the first two generations of quinolones [68]. LEV has been used as a veterinary drug in China, Russia, the United States, Argentina, and India [69]. A previous study revealed that *E. coli* strains isolated from cattle and buffalo farms in Bangladesh were sensitive to LEV [70]. However, 52% (*n* = 44) of *E. coli* isolated from buffalo mastitis samples in Karachi, Pakistan were resistant to LEV [71]. The current study reported that only 5.41% (*n* = 33) of *E. coli* strains were resistant to LEV (Figure 5). At present, no study has systematically evaluated the sensitivity of LEV to buffalo PSM, hence this study serves as the first report. This suggests that we need to carry out targeted research to evaluate the therapeutic effect of LEV in buffalo mastitis.

The *Galleria mellonella* larvae infection test is ideal for evaluating the virulence of pathogenic microorganisms [72,73]. Among the six typical PSM, *E. coli* showed the strongest mortality of *G. mellonella* larvae (Figure 7). *E. coli* is one of the leading pathogens of bovine mastitis, which can cause subclinical and clinical mastitis characterized by systemic changes, abnormal milk appearance, and udder inflammation [74]. *K. pneumoniae* also exhibited a high mortality rate in *G. mellonella* larvae (Figure 7), aligned with the findings of Mai et al. [34]. *K. pneumoniae* contains capsular polysaccharides, lipopolysaccharides, pili, siderophores, and other pathogenic factors [75]. It is an environmental pathogen that can enter the breast through the nipple, causing inflammation of the breast [76,77]. Both *S. chromogenes* (4.76%) and *Staphylococcus epidermidis* (5.55%) exhibited low mortality for *G. mellonella* larvae. Because their pathogenicity is weak in a short period of time, their pathogenicity to *G. mellonella* larvae is persistent. When they undergo long-term and mass reproduction, they will cause serious consequences [78].

## 5. Conclusions

This study investigated the prevalence of PSM in Guangxi, China. A total of 1058 PSM were identified, which were from 95.45% of total samples with coagulase-negative *Staphylococci* (55.30%), *Enterococcus faecalis* (51.52%), *Klebsiella pneumoniae* (28.03%), and *Escherichia coli* (31.82%). All PSM strains showed multiple antibiotic resistance. Levofloxacin may be a suitable antibiotic for the treatment of PSM. *E. coli* and *K. pneumoniae* showed the high mortality of *Galleria mellonella* larvae. In this paper, the pathogenic bacteria, drug resistance, and virulence characteristics of subclinical mastitis in Guangxi buffalo were systematically studied for the first time. In the future, it will be necessary to monitor the emergence of PSM in buffalo farms and develop control strategies to prevent their spread.

## Figures and Tables

**Table 1:** Isolation of major microorganisms (number of total strains isolated ≥ 20).

Bacteria	No. of Total Strains Isolated	No. of Total Samples Containing the Bacteria	Isolation Ratio of Samples (%) ^3^			
			Total Samples *n* = 132	Herd A *n* = 115	Herd B *n* = 8	Herd C *n* = 9
*Enterococcus faecalis* ^1^	386	68	51.52	52.17	-	88.89
*Klebsiella pneumoniae* ^1^	85	37	28.03	32.17	-	-
*Escherichia coli* ^1^	75	42	31.82	24.35	75.00	88.89
*Staphylococcus chromogenes* ^1,2^	59	25	18.94	13.04	25.00	88.89
*Macrococcus caseolyticus* ^1^	59	17	12.88	13.91	-	11.11
*Lactococcus garvieae* ^1^	58	31	23.48	26.09	-	11.11
*Acinetobacter baumannii*	42	22	16.67	19.13	-	-
*Enterococcus gallinarum*	40	23	17.42	19.13	-	11.11
*Enterobacter cloacae* ^1^	33	24	18.18	20.00	-	11.11
*Staphylococcus epidermidis* ^1,2^	31	15	11.36	8.70	-	55.56
*Mammaliicoccus sciuri* ^1^	29	18	13.64	12.17	12.50	33.33
*Staphylococcus haemolyticus* ^1,2^	28	17	12.88	6.96	50.00	55.56
*Streptococcus macedonicus*	25	12	9.09	10.43	-	-
*Kurthia gibsonii*	25	11	8.33	9.57	-	-
*Staphylococcus borealis* ^1,2^	24	14	10.61	6.96	-	66.67
*Aerococcus viridans* ^1^	20	9	6.82	1.74	62.50	22.22
*Staphylococcus cohnii* ^2^	20	10	7.58	8.70	-	-
CoNS	257	73	55.30	49.57	100.00	100.00
PSM	1058	126	95.45	94.78	100.00	100.00
Total	1659	132	100.00	100.00	100.00	100.00

^1^ represents pathogenic bacteria of subclinical mastitis (PSM). ^2^ represents coagulase-negative *Staphylococci* (CoNS). ^3^ Percentage of the number of samples isolated from the strain to the total number of samples.

## Data Availability

The original contributions presented in the study are included in the article/Supplementary Materials. Further inquiries can be directed to the corresponding author.

## Supplementary Materials

The following supporting information can be downloaded at: https://www.mdpi.com/article/10.3390/ani15223321/s1, Table S1: The number of pathogenic bacteria of subclinical mastitis isolated and the basis for judgment; Table S2: Biochemical and 16S rRNA test results of 1659 bacterial strains.

## Abbreviations

The following abbreviations are used in this manuscript:

SCM	Subclinical mastitis
PSM	Pathogenic bacteria of subclinical mastitis
CoNS	Coagulase-negative *Staphylococci*
SCC	Somatic cell count
16S rRNA	16S ribosomal ribonucleic acid
DNA	Deoxyribonucleic acid
PCR	Polymerase chain reaction
S	Sensitive
I	Intermediate
R	Resistant
AMP	Ampicillin
PEN	Penicillin
AMI	Amikacin
GEN	Gentamicin
LEV	Levofloxacin
CIP	Ciprofloxacin
AZI	Azithromycin
CHL	Chloramphenicol
TX	Ceftriaxone
AMOX	Amoxicillin
TET	Tetracycline
TR	Trimethoprim
